# Orthodontic Treatment Completion and Discontinuation in a Rural Sample from North Central Appalachia in the USA

**DOI:** 10.3389/fpubh.2017.00171

**Published:** 2017-07-25

**Authors:** Chris A. Martin, Breana M. Dieringer, Daniel W. McNeil

**Affiliations:** ^1^Department of Orthodontics, West Virginia University School of Dentistry, Morgantown, WV, United States; ^2^Dental Hygiene Program, West Virginia University School of Dentistry, Morgantown, WV, United States; ^3^Department of Psychology, Eberly College of Arts and Sciences, West Virginia University, Morgantown, WV, United States; ^4^Department of Dental Practice and Rural Health, West Virginia University School of Dentistry, Morgantown, WV, United States

**Keywords:** orthodontics, dentistry, Appalachian region, rural, health disparities, treatment completion, adherence

## Abstract

**Background:**

Orthodontics has inherent demands, requiring regular appointments and active patient engagement, but relatively little is established in regard to rates of completion of treatment and possible factors affecting successful completion. These factors may be particularly important for cultural minority groups, such as those in rural Appalachia, given the environmental, social, and economic complexities affecting access to and utilization of treatment.

**Design and methods:**

A naturalistic study design was employed, using retrospective data from a rural outpatient general dental office in July 2012. Chart abstraction yielded 219 (55.3% female) orthodontic patients (M age = 11.0 [3.7]). Chi-square tests for independence were conducted for categorical dependent variables. For continuous variables, *t*-tests were conducted. A logistic multivariate regression analysis was conducted to predict completion/non-completion of treatment, with age, gender, distance traveled, type of malocclusion, and payment type as predictors.

**Results:**

Overall, 49.8% of this sample successfully completed orthodontic treatment. Greater successful conclusion of treatment was found in self-pay patients (i.e., 74%) versus those whose care was funded through Medicaid/Children’s Health Insurance Program (i.e., 34%) or through private insurance (i.e., 36%). Age, gender, and distance to the office from home had no association relative to successful completion of treatment, although average one-way distance to travel for care was considerable (i.e., 38.8 miles).

**Conclusion:**

Rate of successful orthodontic treatment completion was low in this rural sample. Treatment outcome was related to the form of payment for services, with self-pay associated with the highest rate of successful completion.

## Introduction

The utilization of orthodontic treatment by cultural minority groups in the USA is significantly less than that of majority populations (1–4) in spite of treatment need that is at least as great (2, 4). This oral health disparity has important implications for appearance, employability, and self-esteem (5–7), in that lack of access to, and utilization of, orthodontic care is associated with lower oral health quality of life, as well as other psychosocial and economic problems. In the region of Appalachia in the USA, this orthodontic disparity may be particularly pronounced due to a variety of environmental, social, economic, and geographic factors (8, 9). Known as a “neglected minority” (10), the Appalachian population has a number of positive, protective factors, but faces numerous problems and obstacles to good health as well (8, 11, 12).

### Demands of Orthodontic Care and Impact on Adherence and Successful Completion

Orthodontic treatment is unique in that it requires active patient engagement and follow-through on an ongoing basis, typically with regular recall appointments, often at a developmental time of life (i.e., adolescence) in which there is opposition to demands and rules imposed by adults (13). The length of time involved in orthodontic care also is considerable, and can last two or more years, depending on various factors such as the severity of the malocclusion (14).

The classic conceptualization of malocclusion is from Angle, who posited that the relation of the first molars is central, with types including Class I (normal), Class II (lower molars behind the upper molars, causing top teeth to protrude), and Class III (lower molars in front of the upper molars, causing bottom teeth to protrude) (15). Ongoing adherence to oral hygiene, following instructions, wearing removable appliances, and appointment keeping throughout the duration of treatment can be quite challenging, leading to treatment discontinuation and failure.

### Orthodontic Care in Low Income Populations

There are ethnic/racial disparities in utilization of orthodontic care in the USA, with certain minority groups less likely to have had at least one orthodontic visit compared to Whites, in spite of greater problems with malocclusion in some of those groups (1, 2). Access to care is a likely factor, along with various economic and other social issues that are related to both utilization and orthodontic outcomes. Contrasting Medicaid and private-pay patients in Washington state, higher orthodontic completion rates were found among the private-pay patients in a 2-year period; 22% of the Medicaid patients (versus 9% of the others) were judged to have “no improvement” in overall occlusion and esthetics based on standardized clinician assessment (16). Similarly, in Great Britain, lower socioeconomic (SES) status was found to be associated with discontinuing orthodontic care (17), and in the USA was identified as being associated with lower utilization of orthodontics (as was being male) (2). In an investigation of Medicaid-funded orthodontic services in Iowa, children and adolescents living in rural areas and small towns were found to be more likely to utilize orthodontic services relative to those who were urban dwellers (18). A higher rate of appointment failures was found in a sample of Medicaid versus other-pay orthodontic patients at a school of dentistry clinic in Virginia (19).

### Orthodontic Care in Appalachia

A region in the eastern USA that is comprised of 530,948 km^2^ (205,000 miles), Appalachia is shaped by the Appalachian mountain range; as a region, it spans 13 states in the USA, and encompassing all of West Virginia (20). Socioeconomically diverse, much of Appalachia is rural (42% of the population, compared to 20% nationally) and beset by social problems and health disparities (20).

The cultural heritage and values associated with Appalachia include self-reliance, strong religious ties, and loyalty; many groups in Appalachia are composed of peoples who are proud, private, and patriotic, who want to “take care of their own,” and are reluctant to accept charity (11). Lengerich noted that even though this area is faced with limited economic opportunities, and for some, pervasive poverty, many Appalachian communities remain vibrant, and may be a substantial source of its residents’ strength (12).

The mountainous topography shapes lives and culture in Appalachia; access to health care, particularly with specialists, has been hindered by the mountainous terrain, inadequate roads, and transportation systems, and lack of interest of specialists to locate in these areas. This situation continues to demand that patients seek treatment from general health-care professionals (e.g., dentists in general practice) because of lack of access to specialists (e.g., orthodontists), including the distances they would have to travel to receive specialized care.

Research on oral health in Appalachia is growing (21), but as yet only includes a modicum of data on orthodontics. In a sample of 12- to 17-year-old adolescents and their parents in Appalachia, degree of unmet treatment need and history of orthodontic care were similar to the national norms in the youth, although a significant amount of unrecognized and untreated orthodontic need existed in the parents (22). Of additional concern was that demand for orthodontic care among the youth was lower than clinically identified need and less than published norms, which was suggested as possibly being related to oral health values (23). Given the array of oral health issues in Appalachia (21), more information is needed about orthodontic care, given its lifelong implications for occlusion, functionality, and oral health quality of life.

### Objectives and Hypotheses

With reports from practitioners in the field suggesting an alarming rate of orthodontic treatment discontinuation in some population subgroups in Appalachia, this study aimed to document the scope of the problem and to identify possible factors that predict treatment completion and discontinuation. Some prior research has included large datasets from state-based samples [e.g., Ref. (18)], so this study focused on naturalistic data from a single general dental practice in North Central Appalachia. It was hypothesized that successful completion of orthodontics would be related to type of financing for the care [i.e., self-pay, insurance, or government-funded programs including Medicaid and the Children’s Health Insurance Program (CHIP)], with self-pay patients having the highest completion rate. Given the unique nature of the Appalachian sample and environment, as well as oral and other health problems in the region, secondary hypotheses included an array of demographic, orthodontic, and psychosocial factors that the literature has considered in terms of successful completion or possible adverse effects (e.g., promoted discontinuation of treatment), specifically including age at treatment initiation, gender, and distance traveled between home and the office for orthodontic care, and malocclusion type.

## Materials and Methods

A retrospective cohort design was utilized, with data based on existing health records from a rural general dental practice in North Central West Virginia. For a 5-year period (i.e., 2007 through 2012), only records that were completed or inactive for six or more months were included. As this study is a naturalistic one, the only available data were those that already existed in the health records that were utilized in a practice setting.

### Participants and Practice Characteristics

Health records were located for a total of 219 outpatients (121 females, 98 males) with an average age of 11.0 years (SD = 3.7) from a solo private general dental practitioner’s office in rural central West Virginia. The dentist was a general practitioner with training in orthodontics. The office was situated in a rural Appalachian community with a population of approximately 4,100 inhabitants. The county including this community had an estimated population of 16,309, with a 97.9% being Caucasian, 14.1% with a college degree or higher, and 20.6% living below the federal poverty level (24). On the 9-point Rural-Urban Continuum Codes (with 1 = urban and 9 = completely rural), the target county had a rurality status of 7.0, indicating a county with a population of 2,500–19,999, which is not adjacent to a metropolitan area (25).

### Chart Abstraction and Procedure

Data were abstracted from each health record by a single trained dental hygienist (BD) using a standard form. All relevant demographic information available in the chart was recorded (e.g., age at treatment initiation, gender, distance traveled between home and the office for orthodontic care, length of treatment) as was form of payment (i.e., private insurance, Medicaid, CHIP, and self-pay). Also recorded was the Angle’s classification of the occlusion of each patient, as determined by the dentist. Successful completion or non-completion was recorded, and, as applicable, reason for non-completion [i.e., ongoing poor oral hygiene (as determined by the dentist), parent/guardian request, removal by patient].

### Variable Definitions and Statistical Analyses

The primary outcome variable, treatment completion, was created as a dichotomous indicator of whether or not the patient successfully completed treatment (i.e., termination of active treatment with fixed appliances removed by dental staff at the direction of the dentist after a course of treatment that adequately addressed clinical need). Chi-square tests for independence (with Yates Continuity Correction for 2 × 2 analyses) were conducted for categorical dependent variables. For continuous variables, *t*-tests were conducted. Certain variables were combined in particular analyses due to small sample sizes or for clarity of presentation [i.e., Medicaid (*n* = 171) and CHIP (*n* = 5) patients were combined into one group; Divisions 1 and 2 in Class II malocclusion were combined in certain analyses]. To provide an overall perspective on possible determinants of treatment outcome considering possible determinants as a whole, a logistic multivariate regression analysis was conducted to predict completion/non-completion of treatment, with age, gender, distance traveled, type of malocclusion, and payment type as predictors; variables were treated as categorical or continuous, as appropriate.

## Results

Overall, only one-half of all patients successfully completed the prescribed course of orthodontic treatment (i.e., 109 of 219 patients, 49.8%). Reasons for discontinuation included ongoing poor oral hygiene (35.4%, *n* = 39), parent/guardian request (47.3%, *n* = 52), or removal by patient (17.3%, *n* = 19). The distribution of malocclusion across patients was as follows: Class I—15.5% (*n* = 34), Class II (Division 1)—66.7% (*n* = 146), Class II (Division 2)—7.3% (*n* = 16), and Class III—10.5% (*n* = 23).

**Figure 1:**
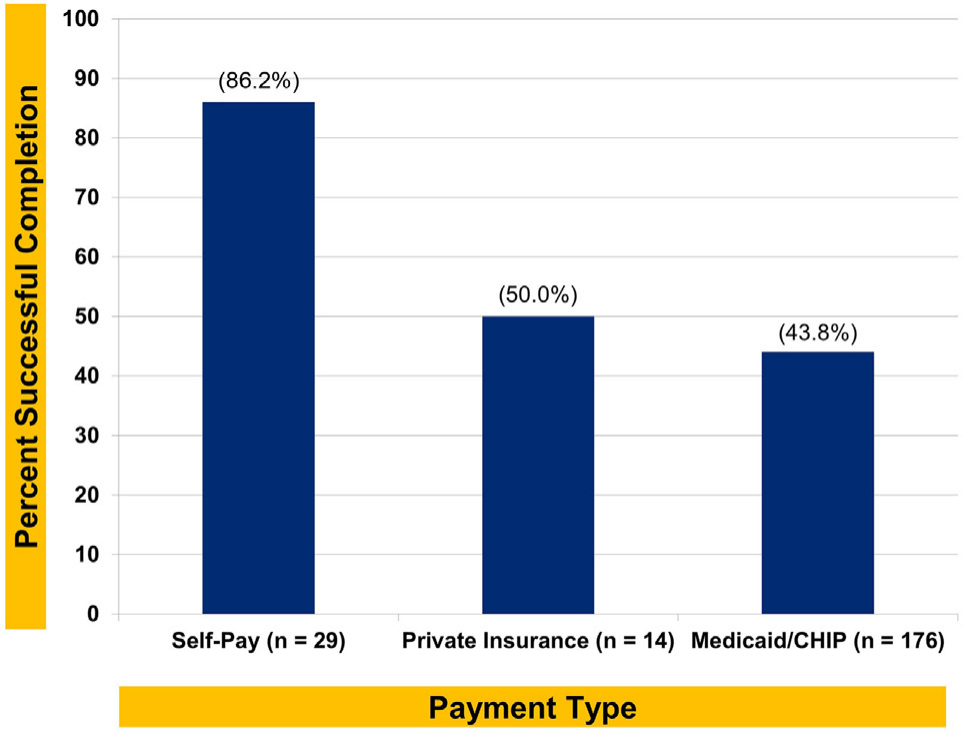
Successful orthodontic completion rate by payment type.

In regard to the first hypothesis, completion rates differed across payment types, χ^2^(2, *N* = 219) = 17.95, *p* < 0.0005; Cramér’s Ѵ = 0.29, *p* < 0.0005. Self-pay patients had a treatment completion rate that was approximately twice that of the other groups, as shown in Figure 1.

Regarding secondary hypotheses, age did not differ between groups [completers: M = 11.4, SD = 4.4; non-completers: M = 10.6, SD = 2.8; *t*(217) = 1.56, *p* = 0.12], nor did gender [completers: males—41% (*n* = 45), females—59% (*n* = 64); non-completers: males—48% (*n* = 53), females—52% (*n* = 57); χ^2^(1, *N* = 219) = 1.10, *p* = 0.31; Cramér’s Ѵ = 0.07, *p* = 0.31]. Distance traveled (one-way) between home and the office for orthodontic care was considerable for some patients (i.e., up to 180 miles; M = 38.8 miles, SD = 32.3), but also did not differ between completer groups, *t*(217) = 0.29, *p* = 0.78. Distribution of type of malocclusion across groups did not differ [completion rate: Class I = 18 of 34, 53%, Class II (Division 1) = 77 of 146, 53%, Class II (Division 2) = 7 of 16, 44%, Class III = 7 of 23, 30%; χ^2^ (1, *N* = 219) = 4.32, *p* = 0.23; Cramér’s Ѵ = 0.14, *p* = 0.23].

The logistic multivariate regression analysis with completion/non-completion of treatment as the dependent variable, and age, gender, distance traveled between home and the office for orthodontic care, malocclusion classification, and payment type as predictors revealed that Medicaid/CHIP and private insurance payment types were significantly related to premature termination of treatment, as indicated in Table 1.

**Table 1:** Logistic multivariate regression predicting treatment completion/non-completion.

Adjusted logistic regression	*B* (SE)	Adjusted odds ratio [95% CI]	*p*
Age	−0.08	0.93 [0.83, 1.03]	0.16
**Gender**			
Male	−0.32	Reference	
Female		0.73 [0.99, 1.01]	0.27
Distance (traveled between home and office)	0.001	1.00 [0.99, 1.01]	0.88
**Malocclusion type**			
Class III		3.39 [0.96, 11.97]	0.058
Class II (both divisions)		0.87 [0.39, 1.94]	0.74
Class I		Reference	
**Payment type**			
Medicaid/Children’s Health Insurance Program		10.08 [3.12, 32.50]	<0.0005
Private insurance		7.55 [1.57, 36.23]	0.012
Self-pay		Reference	

*R^2^ = 0.126 (Cox and Snell), 0.168 (Nagelkerke). Model χ^2^(7) = 29.52, *p* < 0.0005*.

*Premature termination was coded as 1, and successful completion of treatment was coded as 0*.

## Discussion

Payment type was found to be the singular variable that distinguished patients who either did or did not successfully complete orthodontic treatment in this rural Appalachian sample, with an approximately medium effect size (i.e., Cramér’s Ѵ = 0.29) (26). Overall, self-pay patients had a rate (i.e., 86.2%) of successful treatment completion that was twice that of patients whose care was funded by Medicaid/CHIP; privately insured patients had only slightly higher completion rates than the Medicaid/CHIP patients. While payment for orthodontic services through Medicaid may be a proxy for socioeconomic status, with its established relation to health and health behaviors, the fact that completion rates for patients who had private insurance were similar (i.e., 43.8% for Medicaid and 50.0% for private insurance) suggests other influences also affect outcomes.

It should be noted that one of the statistical approaches (i.e., regression) suggested the possibility that Class III malocclusion may be associated with greater premature discontinuation of treatment relative to Class I, although the findings do not reach a standard level of statistical significance. It may be that Class III malocclusion as a condition, with the lower teeth protruding, may be less noticeable and thus less socially compelling for patients to complete treatment.

Cognitive dissonance theory (27, 28) implies that when one invests in a task (e.g., with money, time, or other resources), then one values it to a greater degree, and has more motivation to successfully accomplish it. Patients paying for psychotherapy, for example, tend to benefit more from services than those who are not directly responsible for fees (29). These results prompt consideration of orthodontic payment structures that involve a broad analysis of cost sharing and value for health care, such as in “value-based insurance design” (30, 31). Indeed, behavioral economics approaches have much to offer in terms of improving patient care and practice management in oral health-care settings (32).

These present findings are in many ways similar to those of Mandall and colleagues (33) in a British multi-site study of orthodontic care, who found a 57% completion rate, with poor oral hygiene and multiple failed appointments being the primary reasons for treatment discontinuation. Not dissimilarly, about half of the current Appalachian patients successfully completed orthodontic care, with poor oral hygiene being one of the top three reasons for discontinuation, along with parent/guardian request, and removing appliances at home. Data from a USA urban sample unfortunately are not readily available that would allow comparison with the current findings. Regardless, however, the orthodontic completion rates are quite low, which suggests need for intervention. The present study also found no significant relation between orthodontic outcomes and patient age, gender, or distance between home and the dental office, which is consistent with the Mandall (33) study, although there is some suggestion that Class III malocclusion may be associated with greater premature treatment termination. This latter investigation also noted no relation between orthodontic treatment adherence or orthodontic outcome with demographic factors (including SES), quality of life measures, or clinically determined treatment need (33).

This study is limited in that the sample is from a specific (Appalachian) cultural group in a rural area. At the same time, the sample reflects the ethnic/racial distribution of the region; the unique environmental and social factors affecting this population have implications for other rural and cultural minority groups. Additionally, the sample is limited to a single dental practice in a rural location. Nevertheless, such practices often are isolated, with no specialists available, in rural, low density population areas in Appalachia and elsewhere.

## Conclusion

This study determined that method of payment was related to orthodontic treatment completion, with self-pay patients having twice the rate of successful completion of care, relative to those whose care was funded publically. Consistent with other, international research, there was no relation between treatment completion and demographics, Angle’s malocclusion classification, age, gender, one-way distance traveled for treatment, or length of treatment. Based on 219 orthodontic patients from a general dentistry rural practice in central West Virginia, these results have implications for cultural minority groups receiving orthodontic and other oral health care in rural Appalachia and other rural locales. Given the negative impact of malocclusion on the psychosocial well-being of adolescents (34) and others, and even their academic performance, these findings highlight the importance of developing strategies that will help prevent premature termination of orthodontic care.

## Ethics Statement

This study was carried out in accordance with the recommendations of the American Dental Association Principles of Ethics and Code of Conduct, as well as the American Psychological Association Ethical Principles of Psychologists and Code of Conduct. The protocol was determined to be exempt (protocol #1705579750), as acknowledged by the West Virginia University Institutional Review Board.

## Author Contributions

CM was engaged in study conceptualization, data collection, data analysis/interpretation, and analysis. He provided orthodontic expertise for this project. Additionally, he wrote initial drafts of the paper, and reviewed the final paper and provided editorial comments. BD abstracted the data from the health records, and was engaged in study conceptualization, data collection, data analysis/interpretation, and analysis of results. She reviewed the final paper and provided editorial comments. DM provided expertise in conceptualizing the research questions and hypotheses, as well as research design and analysis. Additionally, he was engaged in all aspects of the current project, leading study conceptualization, data collection, data analysis/interpretation, results writing, and preparation of the manuscript for publication. He edited initial drafts of the paper and wrote the final draft of the paper.

## Conflict of Interest Statement

The authors certify that they have no affiliations with or involvement in any organization or entity with any financial interest (such as honoraria; educational grants; participation in speakers’ bureaus; membership, employment, consultancies, stock ownership, or other equity interest; and expert testimony or patent-licensing arrangements), or non-financial interest (such as personal or professional relationships, affiliations, knowledge, or beliefs) in the subject matter or materials discussed in this manuscript.
